# Observational data reveal evidence and parameters of contagious yawning in the behavioral repertoire of captive-reared chimpanzees (*Pan troglodytes*)

**DOI:** 10.1038/s41598-019-49698-6

**Published:** 2019-09-13

**Authors:** Matthew W. Campbell, Cathleen R. Cox

**Affiliations:** 1 California State University, Channel Islands, California, USA; 2 Los Angeles Zoo and Botanical Gardens, California, USA

**Keywords:** Psychology, Animal behaviour

## Abstract

There is robust experimental evidence for contagious yawning, yet observational studies of naturalistic behavior have been fewer. Without data from real-world behavior, researchers have questioned the existence of contagious yawning and made assumptions about some parameters (e.g., the duration of the effect). We observed contagious yawning in chimpanzees to confirm/disconfirm its existence in the behavioral repertoire of this species, and if present, provide some of the missing descriptives. We recorded yawns on an all-occurrence basis from 18 captive-reared chimpanzees at the Los Angeles Zoo. We recorded identity, time, and individuals who could have been affected. We calculated a threshold for contagion by taking the mean and adding 1.96 standard deviations, constructing a response curve. Across multiple measures we see a consistent pattern in which there is a strong effect of contagion for 1.5 minutes, a less strong but still significant effect lasting up to 3.5 minutes in some measures, and no evidence of contagion beyond 3.5 minutes. From the time stamp on each yawn we were able to rule out temporal synchrony as an alternative hypothesis. Thus, contagious yawning appears to be a natural phenomenon in chimpanzees lending support to the myriad experimental and observational studies to date.

## Introduction

Contagious yawning is when a yawn by one individual induces a yawn in another individual. An assumption of this definition is that the second individual (termed here: the observer) would not have yawned except for the stimulation, visual or auditory, by the first individual (termed here: the trigger). Evidence for the existence of contagious yawning has typically come from experimental approaches: in the most robust designs subjects are exposed to yawns and some other stimulus for control, and rates of yawning in each condition are compared. This approach has yielded consistent, replicated evidence for contagious yawning in humans (*Homo sapiens*)^1–9^ and chimpanzees (*Pan troglodytes*)^10–16^. There is conflicting experimental evidence of contagious yawning in bonobos (*Pan paniscus*), with one study finding an effect^17^ while another did not^12^, but the larger sample size of Tan *et al*.^17^ may explain why they found a significant population-level effect while Amici *et al*.^12^ did not. It is noteworthy that Amici *et al*.^12^ did find a significant effect in chimpanzees, which they had many more of than bonobos. Budgerigars (*Melopsittacus undulates*) have shown experimental^18^ and observational (discussed below) evidence of contagion. Stumptail macaques (*Macaca arctoides*) showed statistically significant contagion in response to video, but it is not clear if the yawning resulted from increased arousal as opposed to copying the expression or affective state of the yawner^19^. Some studies on dogs (*Canis lupus familiaris*) have found evidence of contagious yawning while others have not, which may stem from the different methods used^20–26^. Lastly, two species of lemur (*Lemur catta* and *Varecia variegata*)^27^, lowland gorillas (*Gorilla gorilla*)^12,28^, orangutans (*Pongo abelii*)^12^, and red-footed tortoises (*Geochelone carbonaria*)^29^ did not show evidence of contagion in experimental settings, although the sample size issues mentioned for the bonobo studies also pertain to the orangutans and tortoises.

One issue is that experimental paradigms present subjects with a supernormal stimulus: they encounter more yawns in a shorter amount of time than they would in real life^30^. Anderson^30^ raised the issue of whether contagious yawning is an artifact of experimental designs and is not present in the normal social behavior of the species tested. Taking chimpanzees as an example, despite decades of observational studies in both the field and captivity, no one has reported contagious yawning in their data. Most likely this is because no one has looked for it. As Vick & Paukner^31^ pointed out, previous ethograms of chimpanzee facial expressions did not include yawning. Thus, the lack of contagious yawning in natural behavior that concerned Anderson^30^ could stem from the issue of supernormal stimuli that he raised, but it could just as well stem from researchers not measuring the behavior in the first place, as there are no published data, positive or negative. Whatever the cause, the criticism by Anderson^30^ has thus far not been directly and adequately addressed.

There is some evidence that contagious yawning exists in the normal behavioral repertoires of at least some species. Observational methods have allowed researchers to record yawns of individuals without resorting to playback. These studies found that gelada baboons (*Theropithecus gelada*)^32^, humans^33^, bonobos^34^, and wolves (*Canis lupus*)^35^ were more likely to catch yawns from individuals they had stronger bonds with. The primary goal of these studies was to examine the social relationships of contagion as a means of studying empathy. Three of the four studies also performed some kind of yawn vs. control statistical comparison, which is essential to establishing the existence of contagion^36^. Gelada baboons were more likely to yawn within 5 minutes of perceiving a yawn than other expressions^32^, bonobos who could perceive a yawn were more likely to yawn within 3 minutes than bonobos who could not perceive the yawn^34^, and wolves yawned more within 3 minutes of perceiving a yawn than during a 3-minute matched control^35^. One limitation is that the time window of 3 minutes or 5 minutes is an arbitrary one. There is no empirical evidence that we can find that demonstrates that these are the exact windows of contagion in these, or any, species. Thus, the windows used seem to be judgement calls rather than determined by data.

Using observational methods but a different analysis, budgerigars showed a nonrandom distribution of yawns such that short inter-yawn latencies were more common than would be expected by chance^37^. The benefit of this method is that it lets the data determine the rate of yawning at different time spans post yawn. The authors found a strong effect of yawning within 40 seconds of another yawn, after which the response tapered off. However, it is unclear exactly what the statistical baseline rate of yawning was and when the yawning became statistically indistinguishable from it. Together, these 5 studies using observational methods provide varying support that contagious yawning is not merely an artifact of experimental designs.

The two approaches have resulted in an empirical jump from experimental evidence of the existence of contagious yawning to observational applications of contagious yawning as a means of studying empathy^38,39^. What is missing is a rigorous assessment of the observational evidence of contagious yawning and contagious yawning alone, comparing the rate of contagious yawning to a baseline, and ruling out alternative explanations, like yawns being synchronized over the time of day by similar circadian patterns of activity. An exception is the observational study of budgerigars^37^, which identified a window of contagion (40 s) and ruled out circadian synchrony from their data. The difference in window of contagion between budgerigars (40 s) and those used for mammals (3 or 5 min) is quite large, and it could result from the different methodologies or species differences. Whatever the reason, the budgerigar window of 40 s does not appear to relate well to the mammals that have been studied. Perhaps it is the lack of comparative data that has led some to question the existence of contagious yawning as a natural phenomenon in some species^30^ or at all^40^. Establishing whether contagious yawning exists in behavioral repertoires is important because before we can apply contagious yawning as a method to examine social relationships, we need to know whether it is present in real-world data. If contagious yawning is not present in the daily lives in these species, whether captive or wild, then the applications of contagious yawning as a measure of empathy rests on faulty assumptions. The social relationships in the pattern of contagious yawning may reflect other, unidentified variables, not empathy-based contagion^38,39^.

In this study we sought to identify whether there is statistical evidence of contagious yawning in observational data of a well-established group of captive-living chimpanzees. Our hypothesis was that if yawns are contagious, then there should be a significantly greater chance of a yawn occurring after observing a yawn than the baseline rate of yawning. Rather than selecting an arbitrary time window for contagion, we analyzed all latencies between a yawn and when the individual last saw a yawn and let the data build a response curve. Rates of yawning significantly above the baseline rate of yawning would be evidence of contagion.

In addition to the presence or absence of contagious yawning, our data allow us to describe the parameters of contagion, if it exists. One aspect of contagious yawning that appears different from contagion of other expressions is the extended latency between observing and performing a yawn. With copying other facial expressions (e.g., smiles, frowns, expressions of fear), the window of contagion is 0.5–1.0 seconds^41^. Yawning appears different, however, with an observer yawning some seconds, possibly even minutes, later. This difference between copying yawns and copying other expressions has been enough for some to question the existence of contagious yawning^40^. The window of contagion for yawns is not known for any species other than the budgerigars^37^. Within the mammals, we do not know how quickly one yawn can affect another or for how long as the observational studies used durations that had not been empirically established^32–35^. Our data allow us to measure how soon yawns may affect observer chimpanzees within our temporal resolution. Our data also show how long yawns may be contagious for, but this is only in relation to the population-level rate. Our method is not sensitive to and therefore cannot supply the maximum duration possible for an individual to yawn in response to another.

We also measured some other variables that could affect the rate of contagion, including the number of yawns seen by the observer, the physical proximity to the yawning individual, and the sex of both the observer and the trigger. Since each yawn was time stamped, we examined yawning by time within our 09:30–12:30 observation window. This allowed us to evaluate temporal synchrony (due to internal circadian rhythms or external cues) within our observation times as an alternative hypothesis to contagion if yawns appear to be clustered. In addition, we used the timestamps to examine whether rates of contagion change with time (other studies will need to fill in times for the rest of the day). The group of chimpanzees we studied included 5 infants. Experimental studies have shown an absence of contagious yawning in chimpanzees aged 1–4 years old^10,11^ with emergence beginning at 5 years old^10^. The limitation is that these were experimental studies, and the artificial setting could potentially result in evidence of contagious yawning at an earlier or later age than what occurs naturally. Observational data can reveal when individuals begin to show contagious yawning in their normal social interactions, which could have implications for their social-emotional development. With these data we hope to find out whether statistical evidence of contagious yawning can be identified from observational data, and if so, what its parameters are like.

## Methods

### Ethics

This study adhered to the “Guidelines for the treatment of animals in behavioural research and teaching” developed by the Association for the Study of Animal Behaviour (UK) and the Animal Behavior Society (US)^42^ and all applicable laws. The data collection protocol was approved by the Los Angeles Zoo and Botanical Gardens.

### Subjects

We studied 18 captive-bred chimpanzees (*Pan troglodytes*) at the Los Angeles Zoo and Botanical Gardens. The group consisted of 13 adults (8 female and 5 male, age range: 13–50 years) and 5 infants (3 female and 2 male). All 5 youngsters (age range: 10 months – 5 years 2 months) were observed nursing throughout the study, so all 5 of these individuals are considered infants. The housing consisted of a large outdoor enclosure (3,500 m^2^) which was the main exhibit, indoor sleeping quarters, and a second, smaller outdoor area. Doors controlled access to each. Observations took place in the large outdoor area only. The typical daily routine was for the chimpanzees to be fed around 9:30 (depending upon the husbandry schedule), at which time they would be locked outside on display. The mid-day feeding occurred at 12:30, and the chimpanzees would be let back in for the night around 16:00. Chimpanzees could choose to remain inside in the morning if they wanted, so not all individuals were present in all observations. Once the doors were closed for the morning, there was no further movement inside or out until the end of the day. The chimpanzees had *ad libitum* access to water and food since food from the morning feed was present into the afternoon.

### Observations

All observations took place between 9:30–12:30, though the exact times varied day-to-day. Observations began after the morning feed and ended by the mid-day feed (~12:30). Feeding times were not included in observations. On the rare occasion that supplemental feeding occurred, data collection was suspended while the keepers were present and resumed after they departed.

Observations consisted of all occurrence sampling^43^ of yawns in real time. Data were taken on an iPad Mini (Apple, Inc.) using the program Timestamped Field Notes (Neukadye, Inc.). When a yawn occurred, the researcher (M.C.) recorded the identity of the yawner and any chimpanzees who could have been affected by the yawn. We scored chimpanzees on 3 different levels of potential exposure to the yawn: ‘proximate’ was defined as being within 3 body lengths of the yawner regardless of orientation. We did not require a direct view of the yawn due to the possibility for auditory contagion. ‘Distant view’ was defined as being more than 3 body lengths from the yawner but oriented such that viewing the yawn was probable, and ‘possible view’ was defined as being more than 3 body lengths from the yawner but oriented such that viewing the yawn could have occurred. The difference between ‘distant view’ and ‘possible view’ was the degree of confidence in whether the chimpanzee saw the yawn or not, with ‘distant view’ corresponding to high confidence and ‘possible view’ corresponding to lower confidence. A body length was an estimate based on an average sized adult chimpanzee, splitting the difference between males and females, from head to rump (i.e., not including outstretched arms or legs). Any chimpanzees who did not meet these criteria were assumed to be unexposed to, and therefore unaffected by, the yawn. The program logged the exact time of each entry. In addition, we recorded agonism by the definitions of Romero *et al*.^44^ on an all occurrence basis. As there is a relationship between yawning and anxiety as a displacement behavior^45,46^, yawns occurring within 5 minutes of moderate agonism (contact aggression) by the individuals directly involved in the incident were excluded from the analysis. Bluff displays and non-contact agonism, being of relatively low intensity, did not prompt the exclusion of yawns. We did not attempt to differentiate between yawn types as described by Vick & Paukner^31^, as that required microanalysis from video.

### Criteria and operational definitions

Yawning often occurs in bouts^45^. We defined yawns as belonging to the same bout if they occurred within 5 minutes of another yawn by the same individual. This number is arbitrary, as we have no data from any species about when one bout of yawning ends and a new one begins. It was a judgment call, and 5 minutes seemed reasonable to us. Sometimes in the analysis it is more appropriate to use a count of every yawn, and other times it is more appropriate to analyze bouts. We try to be clear in labeling the analysis as by yawn or by bout.

The problem posed by yawning occurring in bouts can be illustrated with a couple of examples that were both anticipated by us and observed in our data. Scenario 1: If an individual yawns 5 times in short succession, this could be counted as 5 yawns or 1 bout. This matters for contagion because if this individual was induced to yawn by a trigger, then we have the problem of deciding whether this should be rightly counted as one instance of contagion, five instances of contagion, or one contagious yawn followed by 4 noncontagious yawns. Scenario 2: The problem gets even more difficult if two individuals alternate yawning within view of each other. This could be considered multiple instances of contagion as the yawn is transmitted back-and-forth between the individuals, or it could be a single transmission event from trigger to observer. The most conservative and parsimonious approach is to use the smallest number of contagion events that would explain the pattern of yawning, given the bout window we defined of 5 minutes. Therefore, in Scenario 1 we would analyze this as one transmission event. In Scenario 2 all of the yawning by the trigger would be considered to be a noncontagious bout, and the observer’s entire bout would be considered the product of a single point of contagion. The total yawns for each bout in both scenarios would be counted for certain analyses, but the important point is that both scenarios would always be classified as having a single transmission event. This criterion prevents inappropriately inflating the number of contagion events. Future research may show that our concerns were unnecessary, and that situations like Scenario 2 do involve multiple instances of contagion. For now, we feel that our criteria are the safest, most conservative approach to establishing whether contagious yawning can be identified in observational data.

### Analysis

**Figure 1:**
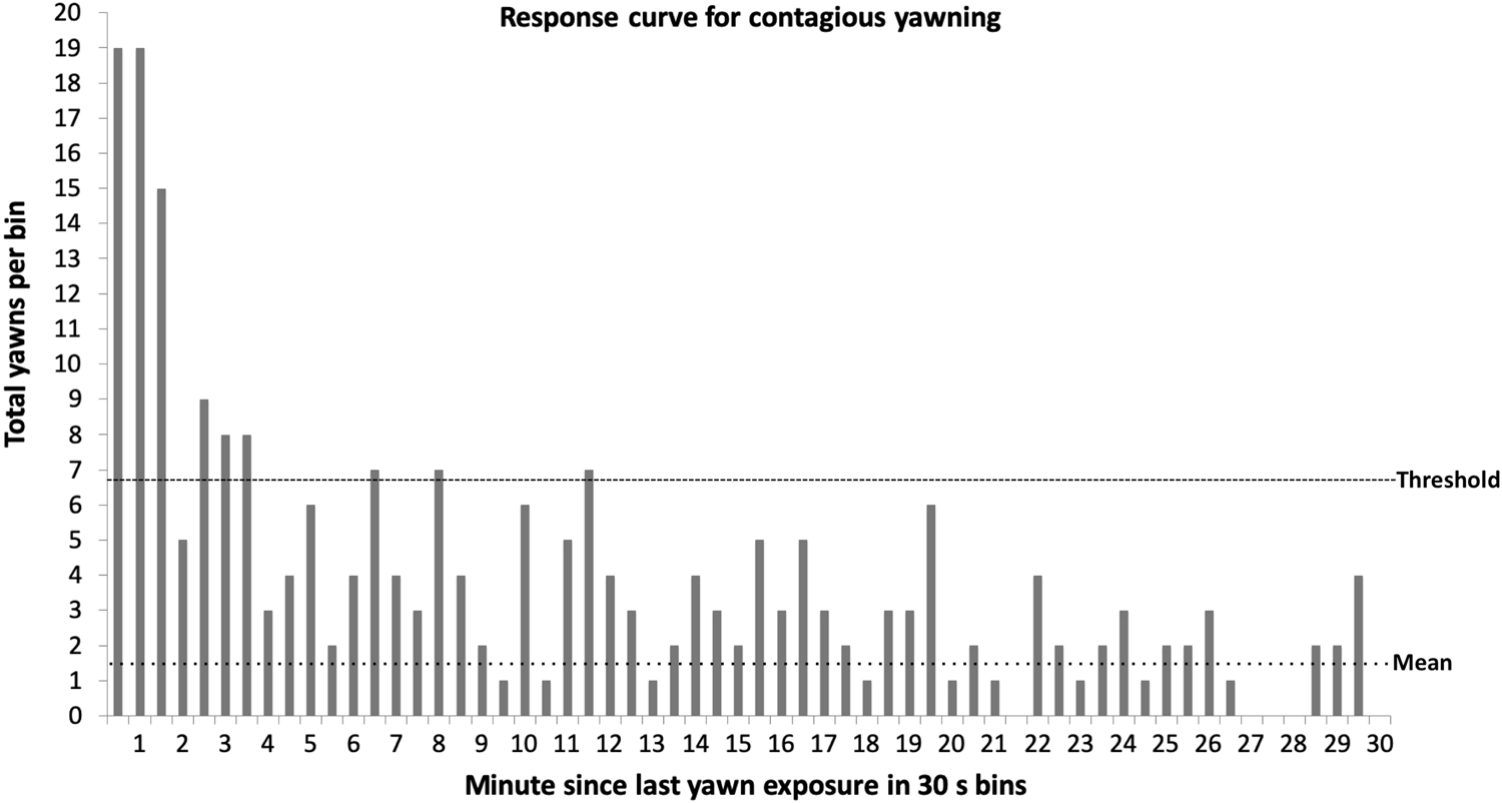
A graph of the number of yawns per 30 second bin. The mean and standard deviation were calculated from all bins (up to minute 100), but we are only showing the first 30 minutes for clarity. Any effect of contagion has diminished well before 30 minutes has passed, and it remains low thereafter. The mean was 1.49 yawns per bin with a standard deviation of 2.66. To establish the threshold we took the mean and added 1.96 times the standard deviation, yielding a threshold of 6.70 yawns per bin (*p* < 0.05 on a *z* distribution). Using this threshold and our criteria for false positives and negatives, we see evidence for contagious yawning starting immediately after viewing a yawn and lasting until 3.5 minutes post yawn. The bin at minute 2 (from 2:00–2:30) is considered “contagious” because both of its neighboring bins are above threshold (criteria for false negatives). The bins at minutes 6.5 (6:00–6:30), 8 (7:30–8:00), and 11.5 (11:00–11:30) are *not* considered “contagious” because they have no neighboring bins above threshold (criteria for false positives).

From this hypothesis we predicted that there should be different response curves based on whether yawns are contagious or not. To establish a response curve, we measured the latency between a yawn and the last time that individual saw a yawn. We then counted the number of yawns for each latency in 30-second bins (e.g., the number of yawns occurring 0–30 s after last seeing a yawn, 30–60 s, 60–90 s, etc., see Fig. 1). From the number of yawns in each bin we computed the mean and standard deviation. To establish a threshold of contagion, we took the baseline rate of yawning (mean) across all bins and added 1.96 standard deviations. This yielded a line above which any rate of yawning would have a less than 5% chance of occurring (i.e., *p* < 0.05) on a *Z* distribution. If yawns are contagious, we expected the rate of yawning to exceed threshold for some duration after observing a yawn. If yawns are not contagious, the rate should remain below the threshold the entire time after observing a yawn. The formulation of this curve is the crux of identifying and describing evidence of contagion from observational data.

#### False positive control

Because we are sampling so many bins (2 per minute with a maximum session of 180 minutes) there is a chance that a bin could be above threshold simply due to sampling error. If a bin is isolated from others above threshold, it would not make sense to conclude that yawns are contagious in these 30 seconds, but not for the time immediately before or after. Thus, to control for false positives we formulated an *a priori* rule that for yawning to be considered contagious in a bin, the rate had to be above threshold and at least one neighboring bin also had to be above threshold.

#### False negative control

There is also the possibility for the reverse; a bin could fall below threshold due to sampling error. If one bin was below threshold within a group of bins above threshold, it would not make sense to conclude that the contagious effect went away during these 30 s and then returned. Thus, to control for false negatives we formulated an *a priori* rule that a bin would be considered contagious even if it was below threshold if both of the neighboring bins were above threshold.

## Results

### Evidence of contagious yawning

We observed the chimpanzees for a total of 169.8 hours across 83 sessions. In this time we recorded 1331 yawns at an overall rate of 0.13 yawns per minute, or one yawn for every 7.65 minutes. These yawns occurred in 792 bouts with an average of 1.68 yawns (SD = 1.15) and a median of 1 yawn (range = 1–10) per bout.

To establish the response curve we analyzed the yawns by bout, so only the first yawn of a bout was used. The response curve is based on the latency since the last yawn exposure, so only yawns in which an individual was previously exposed to a yawn that session were included. If an individual was exposed to more than one yawn by the potential trigger we did not include that yawn because it would not be clear which yawn should be used to calculate the latency. For example, if an observer was exposed to 3 yawns by a trigger, we would not know whether to use the first, middle, or last yawn to calculate the latency. The literature on contagious yawning does not provide an answer, so these yawns were left out of the initial analysis. Likewise, if an observer was exposed to yawns from multiple triggers within a short amount of time (5 min), we do not know which we should use to measure the latency, so these were also excluded from this initial analysis. We were left with 298 bouts. The latencies ranged from 7 s to 1 h 39 m 43 s (median = 11 m 43 s). With a mean of 1.49 yawns per bin and a standard deviation of 2.66, the threshold for evidence of contagion was 6.70 yawns in a bin. Examining Fig. 1 with the criteria described above, we see evidence for contagion starting immediately after seeing a yawn and lasting for 3.5 minutes.

### Levels of exposure

**Figure 2:**
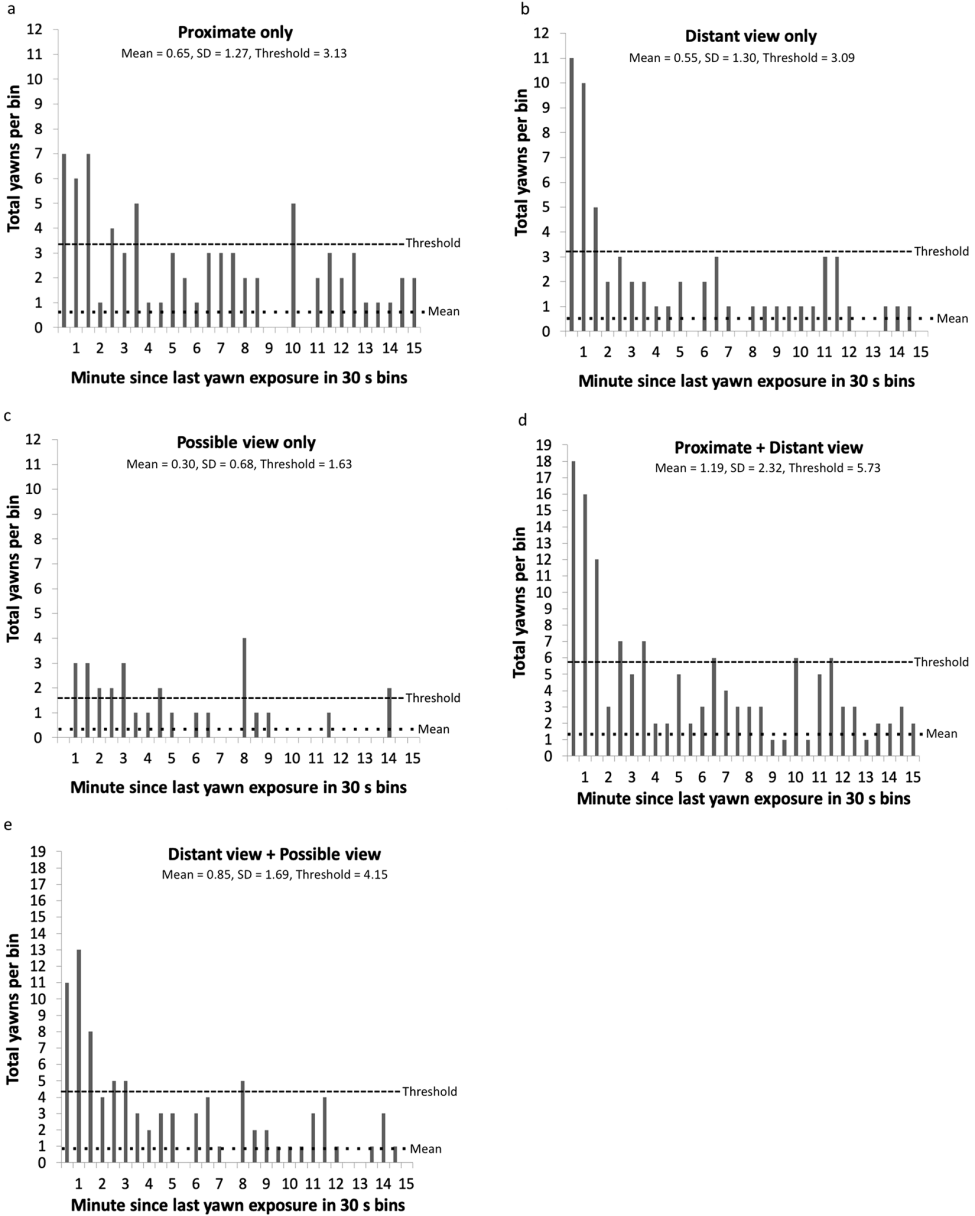
(**a–e**) The number of yawns recorded in each 30 second bin according to the level of exposure. The pattern is similar across all 5 figures with strong contagion through 1.5 minutes, variable evidence for contagion up to 3.5 minutes, and no support for contagion thereafter. For brevity, the figures show the data only up to 15 minutes as there was no evidence for contagion beyond this (Fig. 1).

For each yawn, we recorded how many individuals were exposed to the yawn at each of three levels: ‘proximate,’ ‘distant view,’ and ‘possible view’ (see Methods). We next scrutinized whether these levels were accurate in capturing evidence of contagion. Or, alternatively put, if we do not see evidence of contagion in one of these measures, then maybe it does not accurately measure exposure to yawns (and should therefore not be used in the future). We examined how each level performed by building the same response curve for each level individually. ‘Proximate’ shows strong evidence of contagion for 1.5 min post yawn, and potential continuation until 3.5 min (depending upon how our criteria are applied, Fig. 2a). ‘Distant view’ shows strong evidence of contagion for 1.5 min post yawn with little evidence of contagion thereafter (Fig. 2b). ‘Possible view’ shows an interesting pattern with 0 yawns in the first 30 s, but yawning above threshold from 30 s to 3 min (Fig. 2c). It is not clear whether the lack of yawning in the first 30 s should be interpreted as sampling error (i.e., a false negative) or evidence of a build-up effect in contagion with potentially less strong exposure to a yawn producing contagion at a longer latency. The problem is that the category of ‘possible view’ reflected our confidence in whether the individual was exposed to the yawn or not; it was not intended as a measure of the intensity of the exposure.

To further investigate whether the ‘possible view’ level is a reliable indicator or not, we analyzed only ‘proximate’ and ‘distant view’ together. The results are similar, with strong contagion for 1.5 min and potential contagion until 3.5 min (Fig. 2d). If we leave out the more certain ‘proximate’ level and analyze only the ‘distant view’ and ‘potential view’ together, the results are a similar strong effect for 1.5 minutes, continued above threshold until 3 minutes, and then falling under threshold at 3.5 minutes (Fig. 2e).

### Contagion vs. synchrony

**Figure 3:**
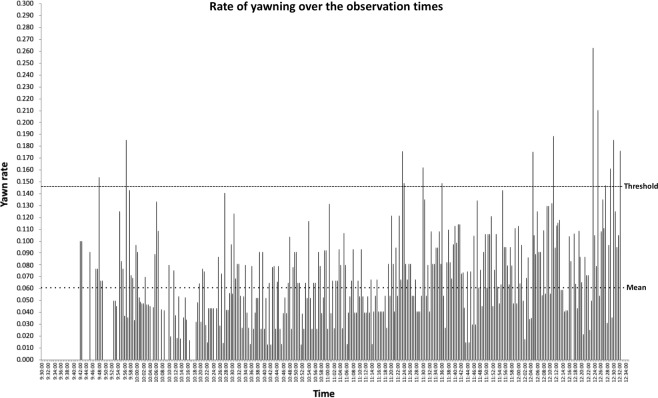
The rate of yawning in 30 second bins throughout the entire range of observation times. We started by taking the time that every yawn recorded was observed and assigning it to a 30 second bin. We then totaled how many times each bin was observed. The number of times a bin was observed varied because our observation times varied due to logistical and husbandry reasons. To calculate the yawn rate, the number of yawns for a bin was then divided by the number of times that bin was observed. The mean rate was 0.061 yawns with a standard deviation of 0.044, yielding a threshold of 0.146. Only two bins meet the criteria for a significant effect, 11:23:00–11:23:30 and 11:23:30–11:24:00, which includes both being above threshold and having at least one neighbor above threshold (control for false positives). These are not enough to explain the 3.5 minute window of elevated yawning we found in Fig. 1.

An alternative hypothesis we need to consider is whether yawns occur closely to each other because they are temporally synchronized, not contagious. Hypothetically, the individuals of the group could proceed through physiological changes on an approximately similar schedule, due to internal mechanisms (a biological clock) or external cues (like feeding times), leading to increases in yawning at some times of the day. This could produce an effect that looks like contagion even though the yawns are all generated independently, rather than being dependent upon perceiving the yawns of others. The first way we assessed the synchrony hypothesis is by formulating a response curve based on the timestamp on each yawn. To do this, we counted how many yawns were observed in 30 s bins (e.g., 9:30:00–9:30:30) from our earliest observation time to our latest. We were not able to observe the exact same times each day due to husbandry and scheduling reasons, which lead to some times of day being observed more than others. To correct for this, we divided the number of yawns by the number of times we observed each bin to give a rate of yawning. We then calculated a mean of 0.061 yawns per bin with a standard deviation of 0.044, yielding a threshold of 0.146 yawns. Looking at all of the bins from our earliest (9:30:00) to our latest (12:34:00) observation time (Fig. 3), we see that only two bins, at 11:23:00–11:23:30 and 11:23:30–11:24:00, are above threshold and have a neighbor above threshold (control for false positives). This 1 minute of potentially elevated yawning is not long enough to explain the 3.5 minutes of elevated yawning in Fig. 1. Nonetheless, we can further examine yawning for effects of temporal synchrony.

### Yawning over the time of the observations

**Figure 4:**
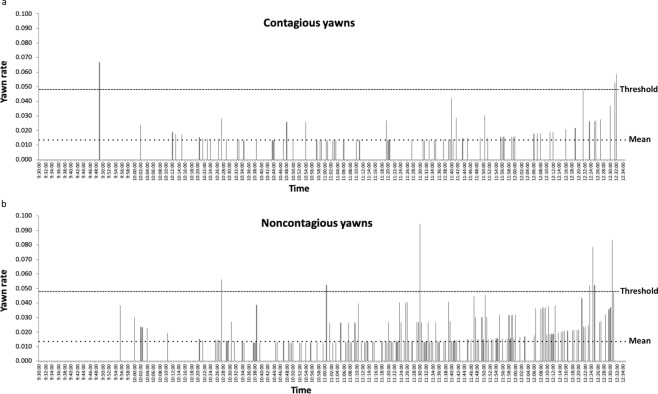
(**a,b**) Response curves for contagious (**a**) and noncontagious (**b**) yawns separately based on time of day. The mean and threshold were calculated from the total data, and then they were applied to contagious and noncontagious yawns separately. The mean rate was 0.013 yawns with a standard deviation of 0.0182, yielding a threshold of 0.0492. For noncontagious yawns, two adjacent bins bins are above threshold at 12:24:00–12:24:30 and 12:24:30–12:25:00. For contagious yawns, two adjacent bins are above threshold at 12:31:30–12:32:00 and 12:32:00–12:32:30. However, all 4 of these bins were viewed very few times (the chimpanzees were fed around 12:30). For the contagious yawns, the yawn rate corresponds to a single yawn in each bin. Therefore, we feel that there is not strong support for the rate of contagion changing with the time of day from the present data. Rather, it is possible that yawning overall increases after 12:00:00.

The timestamp on each yawn allows us to describe yawning across the times that we were able to observe the chimpanzees. To compute this, we used the rate of yawning in each 30 s bin as described in the previous paragraph. Since the data were not normally distributed we used nonparametric correlations. For total yawns, there was a significant, positive, moderate correlation between the rate and time (Spearman’s rho = 0.463, *p* < 0.001), meaning that the yawn rate increased as the observation went on (Fig. 3). We also classified yawn bouts as contagious or noncontagious according to the results in Fig. 1. Contagious yawns were defined as those occurring within 3.5 minutes of an observer viewing a yawn; all others were considered noncontagious. Both contagious (Spearman’s rho = 0.204, *p* < 0.001, Fig. 4a) and noncontagious (Spearman’s rho = 0.466, *p* < 0.001, Fig. 4b) bouts showed significant, positive correlations with the time, but the strength of the correlation is considerably larger for noncontagious yawns. The correlation coefficient for the rate of contagious yawning is considered weak/negligible by convention. Since both the rate of contagious yawning and noncontagious yawning are significantly positively correlated with time, it should be no surprise that they are also correlated with each other, although weakly/negligibly (Spearman’s rho = 0.200, *p* < 0.001). One issue with this correlation is that both samples have a lot of 0’s for the bins (median and mode = 0 for both), and overlap in these 0’s may be driving the correlation. If we remove the 0’s and ask whether a contagious yawn bout in one bin is correlated with a noncontagious yawn bout in the same bin, the correlation is weak and significantly negative (Spearman’s rho = −0.287, p < 0.001). Thus, contagious and noncontagious yawns do not line up across 30 s bins.

**Figure 5:**
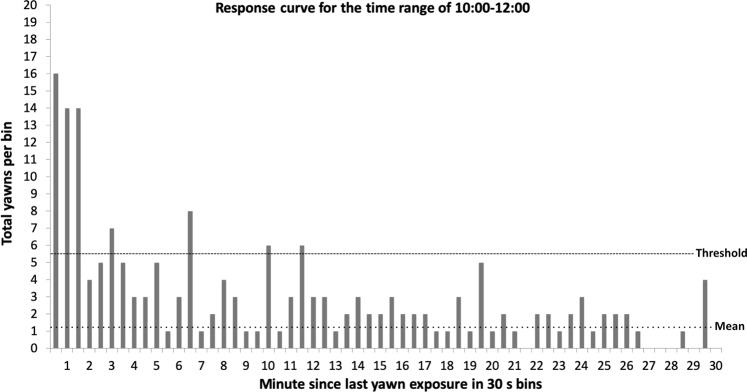
This response curve for contagious yawning was constructed using only the bins from 10:00–12:00 to avoid peaks in yawning associated with earlier and later times in our observation range. The mean and standard deviation were calculated from all bins (up to minute 100), but we are only showing the first 30 minutes for clarity. Any effect of contagion has diminished well before 30 minutes has passed, and it remains low thereafter. With a mean of 1.13 yawns and a standard deviation of 2.19, we calculated a threshold of 5.42 yawns.

Since yawning appears higher at the beginning and end of our observation window, one might argue that the evidence of contagion is solely driven by yawning during these times. That argument would not be consistent with Fig. 4a or the negative correlation ending the previous paragraph, but nonetheless the argument could be made. To scrutinize this, we ran an even more conservative analysis of contagion by only looking at yawning between 10:00–12:00. This 2-hour range avoids the early and late peaks in yawning. Reconstructing our response curve (Fig. 5), we still see strong evidence of contagion for 1.5 minutes post yawn. The rate then drops below threshold before rising above again at 3 minutes, with the bins on either side being just below threshold. After 3.5 minutes the rate drops well below threshold. The pattern is largely similar, even if the rates in the 2.5 and 3.5 minute bins are not quite as strong as seen in Fig. 1.

### Chains of transmission

Observational data allows us to measure aspects of contagion that experiments cannot. One of those is chains of transmission. We examined whether yawns get passed on beyond 2 individuals. Using our conservative measure of contagion, which discarded yawns in which the observer saw multiple yawns by one or more triggers, we recorded 4 instances of triadic contagion in which a yawn was transmitted from a trigger to observer 1, who was then the trigger for observer 2.

Having established evidence for contagion with the most robust effect occurring immediately after viewing a yawn, we can also relax our definition of contagion for the cases where an observer saw multiple yawns by one or more triggers. If we assume that the most recently viewed yawn was the most likely trigger, based on the response curve we established with our conservative criteria (Fig. 1), then our longest chain involved a direct line of 4 transmission events, plus one additional side branch. In this episode, Pandora was the trigger for both Jake and Shaun. The yawning then proceeded from Jake to Jerrard to Glenn to Yoshi. The elapsed time from the assumed triggering yawn until the 5^th^ individual yawned was 9 min 30 s. If we measure all yawns and not just bouts, and if we include Shaun’s side branch that did not get passed on further, this entire episode involved 19 yawns by 6 individuals over 14 min 15 s. Using the relaxed criteria, there is also a 4-chimpanzee chain that circles back to the original trigger (Pandora to Jerrard to Shaun to Pandora). Pandora’s second yawn counts as contagious because it took place more than 5 min after the first, thus the yawns meet our criteria for belonging to separate bouts. This circular chain of transmission lasted 8 min 22 s from the first yawn to the last.

### Further bout analyses

We can use our data to examine whether contagious and noncontagious bouts of yawning differ. The median number of yawns does not differ between contagious and noncontagious bouts (Mann Whitney *U*, N = 792, mean rank noncontagious = 395.65, mean rank contagious = 400.58, *z* = 0.262, *p* = 0.793). Thus, bout length was similar for the two types of yawns. Within bouts of contagion, we can ask whether the distance measure is associated with more or fewer yawns. In other words, do yawns viewed closer (proximate) induce longer bouts than yawns viewed from further away (distant view or possible view). In a sense, this is a measure of potency. There was no correlation between viewing distance and bout length (Kruskal-Wallis *H*_2_ = 0.762, *p* = 0.683). Similarly, yawns caught at a shorter latency (bouts with a latency = 455), might be more potent, leading to longer bouts of yawning in the observer. This was also nonsignificant (Spearman’s rho = −0.035, *p* = 0.457). Lastly, viewing more yawns might raise the chance of contagion. We analyzed this by asking whether contagious bouts had a higher median number of yawns seen than noncontagious bouts, but this was nonsignificant (Mann Whitney *U*, N = 455, mean rank noncontagious = 230.58, mean rank contagious = 222.00, *z* = 0.777, *p* = 0.437).

### Sex differences

To analyze sex differences, we limited the data to the adults only (1284 yawns from 753 bouts). We used Chi squares to compare the observed rate of yawning with the expected rate. The expected rate was calculated on the assumption that the likelihood of yawning was equal for males and females. With 5 adult males in the group and 8 adult females, about 38% (5/13) of the yawns should come from males and 62% (8/13) should come from the females. Using this basis for our expected values, males yawned significantly more than females when analyzed by bout (observed m = 432, f = 321; expected m = 290, f = 463; χ^2^_1_ = 113.08, *p* < 0.001) or by total yawns (observed m = 784, f = 500; expected m = 494, f = 790; χ^2^_1_ = 276.70, *p* < 0.001). In addition, males had significantly more yawns per bout than females (Mann Whitney U, N = 753, mean ranks: m = 395.82, f = 351.67, *z* = 3.128, *p* = 0.002). Males show overall more yawning than females, but this includes both contagious and noncontagious yawns.

Next, we explored whether one sex showed more contagion than the other. To compute this, we first categorized yawns as contagious if they occurred within 3.5 min of viewing a yawn and noncontagious if they occurred more than 3.5 min after viewing a yawn. As expected, males had significantly more noncontagious bouts than females (observed m = 361, f = 259; expected m = 238, f = 382; χ^2^_1_ = 103.17, *p* < 0.001). We then used this rate of noncontagious bouts as the expected values for contagious bouts. In other words, males gave 58% of the noncontagious bouts despite being 38% of the population, and females gave 42% of the noncontagious bouts despite being 62% of the population. Thus, to assess whether either sex showed contagion at a higher rate than the other, we need to use expected values not based on the population size, but rather on the baseline rate of yawning (i.e., noncontagious yawns). Another way of putting this is that since males yawn more than females overall, we would expect more of the contagious yawns to come from males than females. However, that does not tell us whether males *catch* yawns disproportionately to females. To determine susceptibility to contagion we need to use the baseline rate of yawning, not the population size, to calculate the expected values. With expected values based on the baseline rate, there is no difference between the sexes in rates of contagious bouts (observed m = 71, f = 62; expected m = 77, f = 56; χ^2^_1_ = 1.110, *p* = 0.292).

We can also examine the likelihood of inducing contagion in others (i.e., serving as a trigger) from a similar perspective. The most appropriate expected values are based on the baseline rate of yawning for the sexes. Similar to above, since males yawn more than females, there is a greater chance that a contagious bout will have been triggered by a male than a female. That does not tell us whether yawns from males are more likely to be caught than yawns from females, or put differently, whether male yawns are more potent at stimulating contagion than females. With expected values based on the baseline rate, there is no difference between the sexes in triggering yawns in others (observed m = 84, f = 49; expected m = 77, f = 56; χ^2^_1_ = 1.511, *p* = 0.219).

### Development

We recorded 4 yawns from infants that occurred within 3.5 minutes of being exposed to another yawn, and therefore might be considered contagious. These are too few cases to analyze statistically, but we can provide some descriptives. The youngest age of occurrence was 1333 days (3 years, 7 months, 23 days). Three of the four potential contagious yawns occurred when the individual was 3.5–4.0 years old, and one was when the individual was almost 5.0 years old. Both individuals were females, but it is important to note that neither of the young males reached this youngest age (at least 3.5 years old) during the observations. The triggers for all four of these potential contagious yawns were unrelated individuals.

## Discussion

The results show evidence that yawns are contagious in the normal daily life of one captive group of chimpanzees. Our observations allow us to dispel a couple of concerns about contagious yawning. First of all, Anderson^30^ was rightly concerned that experimental studies presented supernormal stimuli to subjects. He raised the question of whether contagion was an artifact of experimental designs, or if it functioned in the normal lives of the animals studied, whether captive or wild. Our results show that contagious yawning does exist in the normal lives of this captive group of chimpanzees, that the effect is strong (>6 standard deviations above baseline during the first minute after viewing a yawn), and that experiments on contagious yawning have probed a natural phenomenon, not an artifact.

Further support that yawns are contagious comes from the chains of transmission analysis. There would be no reason to predict that contagion should stop at only two individuals (one trigger and one observer), and it should be possible for yawns to continue to be transmitted to additional individuals. With our conservative criteria we observed 4 instances of triadic contagion in which yawning proceed from a trigger to observer 1 to observer 2. With a slightly more relaxed definition of contagion, the longest chain moved from a trigger to 4 successive observers. We believe that documenting chains of transmission also supports the existence of contagious yawning as a natural phenomenon.

A second concern is that previous observational studies of contagious yawning did not test for the existence of contagion statistically, as we have done. Three studies chose a window of contagion and found more yawning during that window than baseline^32,34,35^, whereas another did not run an analysis of this sort^33^. The limitation here is that the window of 3 minutes^34,35^ or 5 minutes^32^ was informed by experimental studies, rather than being determined by their own data or other observational studies. Thus, there is some arbitrariness in the choice of times. Only Miller *et al*.^37^ used their data to examine a window of contagion. The problems here are that it is not clear when yawning statistically deviates or returns to baseline from their analysis, the use of one-tailed statistics, and the window identified of only about 40 seconds for budgerigars does not appear to be applicable to the mammals studied.

Because of the issues with observational data, and a lack of it, Anderson^30^ accepted the existence of contagious yawning under experimental conditions and questioned its existence in natural behavior (at least for nonhumans). However, Kapitány & Nielsen^40^ went so far as to question whether contagious yawning exists at all, for any species, including humans. With our results we can dispel the concern of Kapitány & Nielsen^40^ as well. We provide strong statistical support for the existence of contagious yawning in chimpanzees through observational data, which augments the multiple experiments also finding strong statistical support for contagious yawning in this species^10–16^. In addition, our results serve as support for the observational studies that did not test for contagion directly, as we have. The window we identified is similar to ones that have been used previously, and having identified contagious yawning in the behavioral repertoire of two species (chimpanzees in the current study and budgerigars^37^), we expect that similar response curves would be found in the data of others, though the exact numbers may vary by species.

It would also be a mistake to question the findings of the studies that used 5 minutes^32^ or 3 minutes^33–35^ as a window of contagious yawning when our evidence extends to 3.5 minutes. For one thing, our results describe only this group (housed at the Los Angeles Zoo and Botanical Gardens) of this species (*Pan troglodytes*). Our exact numbers may not be indicative of the species as a whole. The group we studied is large by captive standards (N = 18) and diverse (8 adult females, 5 adult males, 5 infants), especially for zoos, but 18 is not enough to establish a species-wide rate of response. In small samples, one or two high functioning or low functioning individuals can skew the precise numbers. The overall pattern should be consistent (yawns are contagious for some time after viewing a yawn), but the exact time window measured might vary between groups. Future testing will be needed to show how representative our parameters of contagion are for chimpanzees as a whole, as well as if they vary at other times of the day.

In addition, there is no reason to expect the parameters we measured to be the same for all species. Different species could have longer or shorter windows of contagion based on their evolution. The other observational studies were on geladas^32^, humans^33^, bonobos^34^, and wolves^35^, and each species may have its own parameters of contagion. We would have to measure them to find out. Lastly, and perhaps most importantly from a statistical perspective, these studies were looking at social patterns of contagion. The expectation was that contagious yawning, if based on empathy mechanisms^38,39^, should be biased toward closer social affiliates than distant ones. Thus, contagious yawning should show a social pattern, but noncontagious yawning should not. Noncontagious yawning should be randomly distributed in the group. Using a window of contagion that is too long would be adding noncontagious yawns (i.e., noise) to the experimental condition, and using one that is too short would be adding some contagious yawns (i.e., signal) to the control condition. If the experimenters still find a significant difference, that implies that the signal is so strong that it can be identified statistically even with extra noise or reduced signal in the input. The implication is that if 5 minutes is too long or 3 minutes is too short and a signal is found, the signal should be even stronger for the optimum window. The problem in including too long a window of contagion is the possibility that the noise (noncontagious yawns) masks the signal (contagious yawns) leading to a null result. With too short of a window there would be a large amount of signal (contagious yawns) in both the contagion condition and the baseline, leading to no significant difference between them. Thus, the problem of using too long or too short of a window of contagion is the risk of a type 2 error (false negative), not a type 1 error (false positive). In our view, our results support the previous observational studies^32–35^ by showing that contagious yawning is present in observational data and do not undermine them by identifying a slightly different and more precise window of contagion.

Unlike previous studies, our results allow us to describe the natural parameters of contagious yawning. We applied numerous different approaches to operationally defining contagious yawning, and the results are remarkably consistent. Using a conservative subset of our data, we found that yawning was contagious for 3.5 minutes after observing a yawn (Fig. 1). Applying different criteria changed the outcome only subtly, and a larger scale pattern emerged with a very strong effect of contagion up until 1.5 min, a less strong but still above threshold effect of contagion after 1.5 min and lasting as long as 3.5 min, and then yawning falling below threshold and staying there. Different measures disagree exactly where the contagion effect falls below threshold (e.g., 1.5 min, 3 min, or 3.5 min), but none extend the window of contagion beyond 3.5 min after viewing a yawn. Since our primary measure of contagion, which we established with a conservative subset of the total yawns, yielded a window of contagion up until 3.5 min, and since this number was supported by other measures, we feel confident in accepting 3.5 min as a duration for which yawns are measurably contagious in this group of chimpanzees. Nonetheless, other researchers may disagree and prefer a more stringent window. The window from using only yawns from 10:00–12:00 to avoid feeding times is tighter, with strong evidence only for the 1.5 minutes post yawn exposure. We have provided our data across multiple criteria for contagious yawning so that readers can come to their own conclusions, based on the evidence we gathered, as to what constitutes the most appropriate window of measuring yawn contagion.

One note, it would be the wrong interpretation to conclude that *all* yawns within 3.5 minutes of viewing a yawn are contagious, and that *no* yawns after 3.5 minutes are contagious. We cannot know for certain, currently, whether any given yawn is contagious or non-contagious. There are no behavioral cues that have been identified to distinguish them, and the only way to determine this conclusively would be through differing neural signatures between yawns originating internally (noncontagious) or through visual or auditory stimulation (contagious). These neural signatures have not yet been identified, and, of course, they would not be helpful outside of laboratory studies. Instead, the window shows the duration of time for which reliable assumptions of pooled data can be made. As done already, the window of the time is the one in which to look for social biases in contagion^32–35^. The window says that a high number of these yawns will be contagious (signal), and that after 3.5 minutes many/most of the yawns will be noncontagious (noise). Nothing says that yawns within 3.5 minutes of view another *must* be contagious. Only that if we are going to look for social patterns, this window provides us with the greatest signal-to-noise contrast with which to do so. In addition, our data do not say that it is impossible for yawns to be contagious beyond 3.5 minutes in chimpanzees. That is also the wrong interpretation. Currently, there is no known difference in the motor pattern between contagious and noncontagious yawns, and one may not exist. Without an observable difference, only a neural method could conceivably label each yawn as contagious or noncontagious, so only that method could establish the absolute maximum duration for which yawns are contagious in any individual or species.

An alternative hypothesis to yawns being contagious is that they are temporally synchronized, perhaps due to circadian rhythms or regular, daily events. Yawning varies over the time of day^45,47,48^. Theoretically, a tight enough peak could produce a pattern that looks statistically like contagion (e.g., resembles Fig. 1) but arises from yawns being temporally synchronized, rather than contagious. However, yawning was not tightly grouped enough in our observations (Fig. 3) to account for the greater chance of observing a yawn after another yawn. The window we measured was only 3.5 minutes long (Fig. 1), at its longest. That would require the temporal pattern of yawning (Fig. 3) to have a similar peak lasting no more than a few minutes. That peak is not present in Fig. 3. Yawning does appear to occur at higher rates at the very beginning and end of our observations, which differs from Vick & Paukner^31^. The increased rates we observed correspond with feedings (just after morning feed and just before lunch), so it is possible that they are displacement behaviors^45,46^ related to tension or anticipation of meal times. Increased yawning around feeding times was also observed in lions (*Panthera leo*) and mandrills (*Mandrillus sphinx*)^47^. However, the times close to the chimpanzees’ feedings (9:30–10:00 and 12:00–12:30) do not correspond exclusively with the time stamps of the contagious yawns we observed (Fig. 4a). Yawns meeting our criteria for contagion appear throughout the observation times, even with a rise toward the end. Furthermore, if we remove these two half-hour ranges and only analyze yawns occurring between 10:00–12:00, we find similar evidence of contagion with a strong effect for 1.5 minutes after viewing a yawn, responses bordering threshold until 3.5 minutes, and then dropping well below threshold thereafter (Fig. 5). Thus, patterns of temporal synchrony cannot explain our data.

The only social data we have in this analysis are the sex and early developmental age of the yawner. Males yawned significantly more than females, which was not recorded in chimpanzees previously^31^. These differences may reflect how a few individuals can influence population-level statistics in the group sizes available in captivity. Larger sample sizes, perhaps from wild groups or sanctuaries, may clarify sex differences in yawning in chimpanzees. Using the higher baseline rate of yawning by males as the expected values, males did not deviate from this rate in their propensity for contagion. In an observational study, female humans showed higher susceptibility to yawn contagion, and the authors linked this difference to females being more empathetic than males in general^49^. We did not make this same observation in chimpanzees as both sexes showed rates of contagion similar to their baseline rate of yawning. Looking at the triggering individual, male chimpanzees^16^ and female bonobos^34^ were more likely to serve as triggers than the opposite sex. However, we did not observe this result, either, as neither sex was more likely to serve as a trigger than expected based on the baseline rate of yawning. Thus, our data do not support either of the previously identified sex differences in contagious yawning. Two of the studies were on different species, so it is possible a female bias in contagion^49^ and triggering^34^ exists in those species, but not in chimpanzees. As for Massen *et al*.^16^, that study was experimental, and ours was observational. One possibility is that the supernormal exposure of video playback^30^ draws out a difference that does not exist in natural behavior. Conversely, the difference may have been too subtle for us to measure with our sample size. The latter seems unlikely because we did not even observe a trend in the direction of males being triggers more often than females. Our observed values match our expected values very closely. Another possibility is that there are differences between the groups of chimpanzees; each study may accurately describe an effect for the group observed, which is not unreasonable when thinking about the diversity of chimpanzee behavior and the relatively small sample sizes of captive groups. Lastly, our study could have a false negative, or Massen *et al*.^16^ could have a false positive. Only replication will solve the discrepancy in these observed effects.

Regarding development, we have too few cases to analyze them statistically, so we need to be cautious with our discussion. We can say that we did record potential contagious yawns from individuals slightly younger than has been seen in experimental studies^10,11^. Anderson *et al*.^11^ used video playback with infants (3 years old) accompanying their parents, and the infants did not yawn at all. The authors did not provide data on attention, so we do not know to what extent the infants perceived the yawns. It is possible the infants were distracted by the presence of their mother, or perhaps the artificiality of video playback does not stimulate contagious yawning at this young age. Madsen *et al*.^10^ used live yawn demonstrations from humans (familiar and unfamiliar) and did not see evidence of contagious yawning under the age of 5 years old. As with Anderson *et al*.^11^, perhaps the artificiality of experimentation fails to elicit behaviors that are developmentally in transition. It is also possible that empathy-based contagion expresses itself sooner for members of the social group, which we can assume would be socially closer to the chimpanzees than members of another species. One last possibility that occurs to us, Madsen *et al*.^10^ studied orphaned chimpanzees, and differences in emotional development have been found between mother-reared and orphaned bonobos^50^. It is interesting that of the 4 potential contagious yawns by infants that we recorded, none of the triggers were the infant’s mother. Because our numbers are so small, we cannot conclude that contagious yawning is in fact present at younger ages than has been recorded previously, but our data do suggest that observational methods may reveal different developmental patterns than experimentation.

In conclusion, we found strong evidence that contagious yawning is a natural phenomenon in a captive group of chimpanzees. We can dispel with the concerns about the very existence of contagious yawning and accept previous experimental and observational studies as measuring aspects of behaviors that exist in the normal repertoires of the species studied. Our methods can be applied widely, and we hope researchers will build response curves for different species. It would be very interesting to know whether the parameters of contagion differ between groups of the same species and across species. Combining the comparative method^51^ and observational studies of contagious yawning could tell us about the evolution of contagious expressions, empathy as a possible mechanism, and the socioecological factors that may have shaped this behavior.

## Data Availability

Our data are available from the corresponding author on reasonable request.
